# Chloroplast Redox Regulatory Mechanisms in Plant Adaptation to Light and Darkness

**DOI:** 10.3389/fpls.2019.00380

**Published:** 2019-04-04

**Authors:** Francisco Javier Cejudo, Valle Ojeda, Víctor Delgado-Requerey, Maricruz González, Juan Manuel Pérez-Ruiz

**Affiliations:** Instituto de Bioquímica Vegetal y Fotosíntesis, Consejo Superior de Investigaciones Científicas, Universidad de Sevilla, Seville, Spain

**Keywords:** chloroplast, hydrogen peroxide, light, darkness, peroxiredoxin, photosynthesis, redox regulation, thioredoxin

## Abstract

Light is probably the most important environmental stimulus for plant development. As sessile organisms, plants have developed regulatory mechanisms that allow the rapid adaptation of their metabolism to changes in light availability. Redox regulation based on disulfide-dithiol exchange constitutes a rapid and reversible post-translational modification, which affects protein conformation and activity. This regulatory mechanism was initially discovered in chloroplasts when it was identified that enzymes of the Calvin-Benson cycle (CBC) are reduced and active during the day and become rapidly inactivated by oxidation in the dark. At present, the large number of redox-sensitive proteins identified in chloroplasts extend redox regulation far beyond the CBC. The classic pathway of redox regulation in chloroplasts establishes that ferredoxin (Fdx) reduced by the photosynthetic electron transport chain fuels reducing equivalents to the large set of thioredoxins (Trxs) of this organelle via the activity of a Fdx-dependent Trx reductase (FTR), hence linking redox regulation to light. In addition, chloroplasts harbor an NADPH-dependent Trx reductase with a joint Trx domain, termed NTRC. The presence in chloroplasts of this NADPH-dependent redox system raises the question of the functional relationship between NTRC and the Fdx-FTR-Trx pathways. Here, we update the current knowledge of these two redox systems focusing on recent evidence showing their functional interrelationship through the action of the thiol-dependent peroxidase, 2-Cys peroxiredoxin (2-Cys Prx). The relevant role of 2-Cys Prxs in chloroplast redox homeostasis suggests that hydrogen peroxide may exert a key function to control the redox state of stromal enzymes. Indeed, recent reports have shown the participation of 2-Cys Prxs in enzyme oxidation in the dark, thus providing an explanation for the long-lasting question of photosynthesis deactivation during the light-dark transition.

## Introduction

Photosynthesis is the process that allows the use of light energy for biomass production using water as source of reducing power; hence, chloroplasts have the function of providing the metabolic intermediates that support plant growth. These intermediates include molecules with signaling function such as hormones and hormone precursors, so that chloroplast performance also plays an important role in the harmonization of the growth of the different organs during all stages of plant development. Therefore, light is a key environmental factor for plant growth and development. Some of the changes in light availability, i.e., intensity and quality, vary in a predictable manner and plants, like other organisms, are able to anticipate these changes by the circadian clock. However, in nature, light availability changes continuously and, consequently, chloroplast performance needs to be rapidly adapted to these unpredictable conditions.

Central for the ability of chloroplast metabolism to rapidly respond to changes in light intensity is thiol-based redox regulation, which relies on the extraordinary properties of the thiol group of cysteines (Cremers and Jakob, 2013). Considering random distribution of amino acids in proteins, cysteine is underrepresented in all organisms though its presence appears to correlate with complexity ranging between 2.26% in mammals to 0.5% in some archaebacteria (Miseta and Csutora, 2000). The thiol group of cysteines is very sensitive to oxidant conditions, being able to react with hydrogen peroxide so that it may be oxidized as sulfenic (-SOH), sulfinic (-SO_2_H), and even sulfonic acid (-SO_3_H) (Cremers and Jakob, 2013). In its reduced form, cysteine may react with another cysteine forming a disulfide bridge. Cysteine residues involved in dithiol-disulfide redox exchange usually show a high degree of conservation in redox-sensitive proteins, the conformation and activity of which is deeply affected by the redox state of these pairs of cysteines.

**Figure 1:**
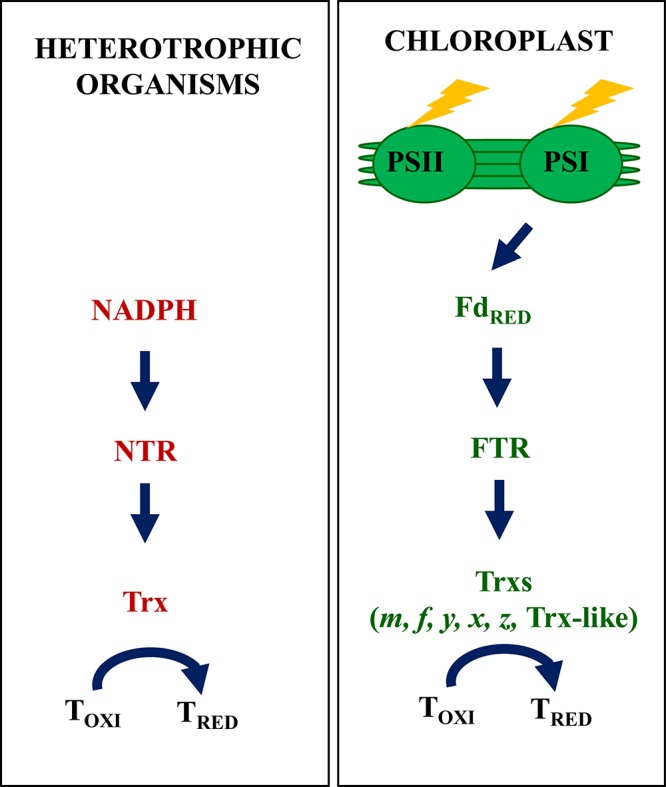
The classic view of redox regulatory pathways in heterotrophic organisms and plant chloroplasts. Regulation of enzyme activity based on the disulfide-dithiol exchange is a universal mechanism present in any type of organisms from bacteria and fungi to animals and plants. In heterotrophic organisms, this regulation is largely based in a two-component system. The disulfide reductase activity of Trx catalyzes the reduction of regulatory disulfides in target proteins (T), the required reducing power being provided by NADPH via the participation of NTR. In plant chloroplasts redox regulation is essential for the rapid adaptation of metabolism to ever changing light availability. Chloroplasts harbor a complex set of Trxs and Trx-like proteins, which rely on photo-reduced Fdx via the participation of FTR, thus linking redox regulation of enzyme activity to light. In addition, chloroplasts contain an additional redox system, NTRC, which relies on NADPH (see below, Figure 2).

Dithiol-disulfide exchange constitutes a universal regulatory mechanism present in all types of organisms from bacteria and fungi to plants and animals. The reduction of disulfide bridges in redox-regulated proteins relies on the protein disulfide reductase activity of thioredoxins (Trxs) and glutaredoxins (Grxs) (Meyer et al., 2012). However, most studies in different types of organisms have focused on the regulatory properties of Trxs, small polypeptides of 12–14 kDa with a well-conserved active site (WCGPC), which were initially described as cofactors of ribonucleotide reductase from *Escherichia coli* (Laurent et al., 1964). In heterotrophic organisms, the reducing power required for Trx activity is provided by NADPH via the action of an NADPH-dependent Trx reductase (NTR) (Jacquot et al., 2009; Figure 1). Interestingly, despite the large number of proteins that undergo redox regulation in heterotrophic organisms, the gene families encoding NTRs and Trxs is rather low, having typically two, at most three, members (Meyer et al., 2012). Redox regulation is also very important in plant chloroplasts; however, in these organelles, this regulatory mechanism shows remarkable differences as compared to heterotrophic organisms. In this review, we discuss the complex redox regulatory network of plant chloroplasts, focusing on the relevance of redox regulation for the rapid adaptation of chloroplast metabolism to light availability.

## Thiol-Dependent Redox Regulation in Plant Chloroplasts

Redox regulation of enzyme activity was first discovered by the seminal work of Prof. Buchanan at Berkeley (Buchanan, 2016). The initial key finding was that photo-reduced Fdx is required for the activation of the Calvin-Benson cycle (CBC) enzyme fructose bisphosphatase (FBPase) (Buchanan et al., 1967). Further analyses uncovered the participation of two components, Trx and an Fdx-dependent Trx reductase (FTR), which fuel reducing equivalents from photo-reduced Fdx for the reduction of a regulatory disulfide of FBPase, which rendered a more active form of the enzyme (Buchanan et al., 1971; Wolosiuk and Buchanan, 1977). Different approaches based on biochemical analyses soon identified two isoforms of Trxs in chloroplasts, *f* and *m*, named on their ability to preferentially activate FBPase and NADP-malate dehydrogenase, respectively (Buchanan, 2016). More recently, the enormous availability of genome sequence data from a large number of plant species has shown that chloroplasts harbor a complex set of up to twenty Trxs and Trx-like proteins (Buchanan et al., 2012; Meyer et al., 2012; Balsera et al., 2014; Geigenberger et al., 2017). In addition, mass spectrometry analyses in conjunction with techniques for trapping proteins interacting with Trxs have allowed the identification of a large number of putative Trx targets from chloroplasts (Montrichard et al., 2009). Although the redox regulation of many of these proteins awaits *in vivo* experimental confirmation, the number of putative Trx-interacting enzymes so far identified suggests that redox regulation may virtually affect any process occurring in the chloroplast, being thus a regulatory mechanism essential for the rapid adaptation of chloroplast performance to ever changing light conditions.

The classical view of redox regulation in chloroplasts, based on the FTR-Trx system, relies on Fdx reduced by the photosynthetic electron transport chain, which thus directly links redox regulation in the organelle to light (Schürmann and Buchanan, 2008). This is in clear contrast with heterotrophic organisms, where redox regulation relies on NADPH and the NTR-Trx redox system (Buchanan, 2017). Therefore, a major difference in redox regulation between chloroplasts and heterotrophic organisms is the source of reducing power used for this regulatory mechanism (Figure 1). This notion was modified by the discovery of NTRC, a novel NTR with a joint Trx domain at the C-terminus (Serrato et al., 2002, 2004). NTRC is exclusively found in organisms that perform oxygenic photosynthesis, namely plants, in which it is encoded by one or two genes, algae and some, but not all, cyanobacteria (Pascual et al., 2010; Nájera et al., 2017). In plants, NTRC shows localization in all types of plastids, either from photosynthetic or non-photosynthetic tissues (Kirchsteiger et al., 2012); however, it is a relatively abundant protein in chloroplasts where it shows stromal localization (Serrato et al., 2004; Moon et al., 2006; Pérez-Ruiz et al., 2009). Initial biochemical analyses of NTRC, based on the study of truncated polypeptides containing the NTR or the Trx domain of the enzyme, confirmed the NTR and Trx activities, respectively, of these domains (Serrato et al., 2004). Thus, based on these results, we suggested that NTRC is a bi-functional enzyme, which might function either as NTR or as Trx in the chloroplast. Soon afterward, it was reported that NTRC is able to efficiently reduce 2-Cys peroxiredoxins (2-Cys Prxs) (Moon et al., 2006; Pérez-Ruiz et al., 2006; Alkhalfioui et al., 2007). Indeed, the incubation of NTRC and 2-Cys Prx allows the rapid reduction of hydrogen peroxide using NADPH as source of reducing power, hence showing that the enzyme is able to conjugate both NTR and Trx activities for the efficient reduction of these thiol-peroxidases. Further biochemical analyses using mutated versions of the enzyme suggested that the catalytic active form of NTRC is a dimer arranged in a head-to-tail conformation, which interacts with 2-Cys Prxs through the Trx domain (Pérez-Ruiz and Cejudo, 2009; Bernal-Bayard et al., 2012). Overall, these evidences uncover the use of NADPH to support the antioxidant function of 2-Cys Prxs in chloroplasts (Pérez-Ruiz et al., 2006). It is known that 2-Cys Prxs, which are among the most abundant proteins of the chloroplast stroma (Peltier et al., 2006), show a very efficient hydrogen peroxide scavenging activity (Dietz, 2011; Perkins et al., 2015; Liebthal et al., 2018). In addition to NTRC, chloroplast Trxs and Trx-like proteins also show capacity of 2-Cys Prx reduction *in vitro* (Broin et al., 2002; Collin et al., 2003, 2004; Dangoor et al., 2012; Eliyahu et al., 2015; Hochmal et al., 2016; Vaseghi et al., 2018; Yoshida et al., 2018). *In vitro* assays comparing several chloroplast Trxs indicated that type-*x* Trx is the most efficient 2-Cys Prx reductant (Collin et al., 2003). The comparison of the *in vivo* redox state of 2-Cys Prxs in NTRC and Trx *x* Arabidopsis knock out mutants showed a severe impairment of the redox state of the 2-Cys Prxs in the *ntrc* mutant, whereas in the *trxx* mutant it was indistinguishable of the wild type (Pulido et al., 2010). Based on these results, it was proposed that NTRC is the most relevant reductant of 2-Cys Prxs *in vivo*. Because NTRC interacts with 2-Cys Prxs by the Trx domain, it could be considered that NTRC is a Trx that incorporates its own reductase, which would explain the high catalytic efficiency of this enzyme (Cejudo et al., 2012). However, the presence of an NTR domain in NTRC raises the question of whether this enzyme also enables the transfer of reducing equivalents from NADPH to plastidial Trxs. The overexpression of the NTR domain of NTRC in the Arabidopsis *ntrc* mutant partially recovered the wild type phenotype, suggesting that NTRC displays NTR activity and might interact with chloroplast Trxs, in particular with Trx *f* (Toivola et al., 2013). This notion, however, is questioned by the fact that NTRC is unable to reduce neither of the chloroplast Trxs *in vitro* (Bohrer et al., 2012). Therefore, whether NTRC interacts with its targets exclusively through the Trx domain, as it does with 2-Cys Prxs, or through the NTR domain, as it was proposed for Trx *f*, remains an open issue.

## The Chloroplast Redox Systems NTRC and Fdx-FTR-Trx Act Concertedly

The identification of NTRC as an NADPH-dependent redox system established the presence of two redox pathways in chloroplasts, hence raising the question of the functional relationship between them. This issue is being addressed through reverse genetic approaches in *Arabidopsis thaliana*. The deficiency of different chloroplast Trxs results in a surprising low effect on plant growth. This is the case of knock out mutants for Trx *x* (Pulido et al., 2010) or Trxs *y* (Laugier et al., 2013), which show growth phenotypes very similar to the wild type. More intriguingly, despite the major role proposed for Trxs *f* in light-dependent redox regulation of CBC enzymes (Michelet et al., 2013), the double knockout mutant of Arabidopsis lacking Trxs *f*1 and *f*2 show almost wild type growth phenotype (Yoshida et al., 2015; Naranjo et al., 2016a). An in-depth analysis of the *in vivo* redox state of FBPase in this mutant revealed that the light-dependent redox regulation of the enzyme was only partially affected (Naranjo et al., 2016a), indicating that other chloroplast Trxs contribute to the redox regulation of FBPase. In this regard, it was shown that *m*-type Trxs have a relevant function in the light-dependent redox regulation of CBC enzymes (Okegawa and Motohashi, 2015). There are four isoforms of type *m* Trxs in Arabidopsis (Okegawa and Motohashi, 2015). Single mutants deficient in Trxs *m*1 and *m*4 show growth phenotypes similar to the wild type (Courteille et al., 2013; Laugier et al., 2013), though the deficiency of Trx *m*4 causes up-regulation of the NADH dehydrogenase-like complex-dependent plastoquinone reduction pathway of photosynthetic electron transport (Courteille et al., 2013). Notably, mutant plants devoid of Trx *m*3, the less abundant *m*-type Trx (Okegawa and Motohashi, 2015) showed unaffected chloroplast performance but impaired symplastic trafficking (Benítez-Alfonso et al., 2009). To our knowledge, no mutants completely devoid of the four *m*-type Trxs have been reported, however, approaches based on gene silencing generated Arabidopsis plants with very decreased contents of Trxs *m*1, *m*2 and *m*4, which allowed to propose a function of these *m*-type Trxs in photosystem II (PSII) biogenesis (Wang et al., 2013). Plants impaired in the expression of the variable subunit of FTR, which provides electrons to plastidial Trxs, display marked phenotype traits such as sensitivity to oxidative stress, impaired light-dependent reduction of Trx-regulated enzymes and increased contents of 2-Cys Prxs (Keryer et al., 2004). The latter trait giving further support to the tight relationship between FTR and NTRC redox systems. Finally, it should be mentioned that the deficiency of Trx *z* affects chloroplast transcription, thus compromising chloroplast biogenesis (Arsova et al., 2010). However, it is not clear whether the role of Trx *z* in the expression of plastid-encoded genes is redox-dependent (Wimmelbacher and Bornke, 2014).

In clear contrast, the Arabidopsis NTRC knockout mutant, *ntrc*, shows a characteristic phenotype consisting in retarded growth and pale green leaves, with about 70–75 % of the chlorophyll contents of the wild type plants (Serrato et al., 2004; Lepistö et al., 2009). Interestingly, the growth phenotype of the *ntrc* mutant is highly dependent on light availability; i.e., growth retard is aggravated when plants are grown under short-day conditions, as compared with long-day conditions (Pérez-Ruiz et al., 2006; Lepistö et al., 2009), and fluctuating light intensities (Thormählen et al., 2017). In addition, the Arabidopsis *ntrc* mutant shows high sensitivity to different abiotic stresses including high salt (Serrato et al., 2004), prolonged darkness (Pérez-Ruiz et al., 2006), and high temperature (Chae et al., 2013), as well as to biotic stress (Ishiga et al., 2012, 2016). As 2-Cys Prxs are very efficient hydrogen peroxide scavengers and NTRC is the most efficient reductant of these enzymes, the increased sensitivity of the *ntrc* mutant to biotic and abiotic stresses might be in agreement with the antioxidant function proposed for the enzyme. Therefore, the antioxidant function proposed for NTRC implies that NADPH serves as source of reducing power in chloroplasts, at least to support the hydrogen peroxide scavenging activity of 2-Cys Prxs (Spínola et al., 2008). This notion not only suggested a relevant role for NADPH in chloroplast redox homeostasis, it changes the paradigm of redox regulation linked to light, as NADPH is produced in chloroplasts from photo-reduced Fdx but also from sugars by the oxidative pentose phosphate pathway during darkness.

**Figure 2:**
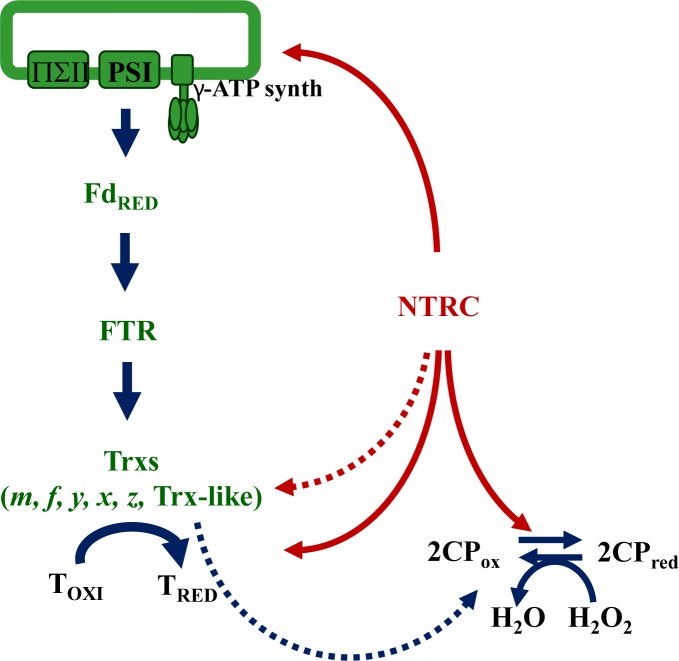
NTRC allows the use of NADPH in chloroplast redox homeostasis. NTRC is an efficient reductant of 2-Cys Prxs and, thus, allows the use of reducing equivalents from NADPH to support the hydrogen peroxide scavenging activity of these thiol-dependent peroxidases. Though at lower efficiency, chloroplast Trxs are also able to reduce 2-Cys Prxs. Different studies have shown the participation of NTRC in the upstream photochemical reactions of photosynthesis and the redox regulation of the γ subunit of ATP synthase (γ), and of downstream targets including enzymes of the Calvin-Benson cycle, starch and chlorophyll biosynthesis. Moreover, NTRC might display NTR activity reducing chloroplast Trxs such as Trx *f*.

In summary, the approaches based on the analysis of Arabidopsis mutants show that while the deficiency of NTRC causes a severe effect on plant growth and development, the deficiency of different types of chloroplast Trxs, with the exception of Trx *z*, has low or no effect on plant growth, indicating the functional redundancy of these enzymes. Moreover, the fact that the absence of NTRC has such severe phenotype suggests the participation of this enzyme in different aspects of chloroplast redox homeostasis, besides its proposed antioxidant function as electron donor to 2-Cys Prxs. Key chloroplast metabolic pathways, such as chlorophyll and starch biosynthesis, and light energy utilization, which were known to be regulated by the canonical FTR/Trx pathway, are also affected by NTRC. A first indication of the effect of NTRC on redox-regulated processes was the finding that the pathway of chlorophyll biosynthesis is impaired in the *ntrc* mutant (Stenbaek et al., 2008). Initially, it was proposed that the positive effect of NTRC is exerted by the reduction of 2-Cys Prxs, which protects the aerobic cyclase activity of oxidant conditions. Further analyses revealed a direct role of NTRC on the redox regulation of the chlorophyll biosynthesis enzyme MgP methyltransferase (CHLM) and direct interaction of NTRC with CHLM and glutamyl-transfer RNA reductase1 (GluTR1) (Richter et al., 2013). Moreover, the content of both CHLM and GluTR enzymes were decreased in the *ntrc* mutant, suggesting that NTRC also affects the stability of these enzymes (Richter et al., 2013). In line with this observation, the I subunit (CHLI) of Mg chelatase, a redox-regulated enzyme that catalyzes the first dedicated step of chlorophyll biosynthesis (Ikegami et al., 2007), was shown to be regulated by NTRC (Pérez-Ruiz et al., 2014). Taken together, these results strongly suggest the participation of NTRC in the redox regulation of the chlorophyll biosynthesis pathway. In addition, NTRC participates in the redox regulation of starch biosynthesis (Michalska et al., 2009). A key regulatory step of this pathway is catalyzed by ADP glucose pyrophosphorylase (AGPase), a heterotetrameric enzyme formed by two small and two large subunits. Under illumination, AGPase is activated by the reduction of a disulfide bridge that links the two small subunits, a process that was described to be regulated by Trxs (Geigenberger, 2011). Indeed, Arabidopsis mutant plants deficient in Trx *f*1 show decreased light-dependent activation of AGPase (Thormählen et al., 2013). Interestingly, the Arabidopsis *ntrc* mutant shows decreased starch contents in leaves, and impaired reduction of AGPase in response to light (Michalska et al., 2009). Moreover, NTRC interacts and triggers the reduction of the enzyme *in vitro*, thus indicating the participation of NTRC in the light-dependent reductive activation of AGPase (Michalska et al., 2009), a notion further confirmed by the analysis of the regulation of starch metabolism in the *ntrc* mutant grown under short-day conditions (Lepistö et al., 2013). Altogether, these studies show that redox regulation of chlorophyll and starch biosynthesis, two key metabolic pathways previously shown to be regulated by Trxs, are also regulated by NTRC, indicating overlapping functions of the FTR-Trxs and NTRC redox systems in plant chloroplasts. Finally, the analysis of photochemical parameters revealed that mutant plants lacking NTRC show high non-photochemical quenching (NPQ) (Thormählen et al., 2015; Carrillo et al., 2016; Naranjo et al., 2016b). Although the *ntrc* mutant shows altered xanthophyll cycle (Naranjo et al., 2016b) and impaired reduction of the γ subunit ATP synthase under low irradiance, which causes higher lumen acidification and lower electron transport rate (Carrillo et al., 2016), the ultimate reason to explain how NTRC affects the efficiency of light energy utilization remains unknown. Nevertheless, these results reveal that NTRC does not only participate in the light-dependent redox regulation of metabolic pathways including the biosynthesis of starch and chlorophyll, it does also participate in the regulation of photochemical reactions such as NPQ. Therefore, the action of NTRC in chloroplast performance is exerted by the antioxidant function of the enzyme due to its capacity to reduce 2-Cys Prxs, but also on upstream photochemical reactions and on downstream redox-regulated targets (Figure 2).

A useful approach for understanding the functional relationship between the FTR-Trxs and NTRC redox systems was the analysis of Arabidopsis mutants simultaneously deficient in both pathways. While mutant plants knock out for Trx *f*1 (Thormählen et al., 2013), or Trxs*f*1 and *f*2 (Yoshida et al., 2015; Naranjo et al., 2016a) showed slight growth phenotype effects, the combined deficiencies of Trxs *f* and NTRC, the *ntrc-trxf1f2* mutant, produced a very severe growth inhibition phenotype (Thormählen et al., 2015; Ojeda et al., 2017). The dramatic growth inhibition phenotype of these mutants is in agreement with the severe decrease of light energy utilization efficiency (Ojeda et al., 2017). Similarly, the lack of Trx *x* has almost no effect on plant growth (Pulido et al., 2010), whereas the simultaneous deficiency of Trx *x* and NTRC causes as well a dramatic effect of plant growth, much more severe than that of the *trxx* or *ntrc* single mutants (Ojeda et al., 2017). Interestingly, a remarkable feature of the *ntrc-trxf1f2* and the *ntrc-trxx* mutants is the high mortality at the seedling stage (Ojeda et al., 2017), suggesting that chloroplast redox regulatory mechanisms are essential at the cotyledon-to-true leaf transition, which is a critical stage of plant development. The fact that the lack of chloroplast Trxs causes such a dramatic effect when combined with the lack of NTRC was further supported by the finding that mutant plants simultaneously devoid of NTRC and the catalytic subunit of FTR are inviable (Yoshida and Hisabori, 2016).

It has been reported that NTRC interacts with well-established Trx targets such as phosphoribulokinase (PRK) and FBPase (Nikkanen et al., 2016). Moreover, the analysis of the redox state of FBPase, a well-known redox-regulated enzyme of the CBC, showed decreased level of light-dependent reduction in the *ntrc* (Ojeda et al., 2017), and the *trxf1f2* (Naranjo et al., 2016a) mutants. Intriguingly, the level of light-dependent reduction of FBPase in the *trxx* and the *trxf1f2* mutants was very similar (Ojeda et al., 2017), despite the fact that Trx *x* is considered not involved in the redox regulation of enzymes of the CBC (Collin et al., 2003). The light-dependent redox reduction of FBPase is even more affected in the *ntrc* mutant, and essentially undetectable in mutant plants simultaneously lacking Trxs *f* or *x* and NTRC (Ojeda et al., 2017). Thus, these results support the notion that both Trxs (*f* and *x*) and NTRC concertedly participate in the redox regulation of FBPase. A possibility to explain the concerted action of the NTRC and the FTR-Trxs redox systems is that both have overlapping regulatory activity on the different redox regulated targets. Remarkably, *in vitro* assays with the purified proteins confirmed the high efficiency of Trxs *f*1 and *f*2, as compared with Trx *x*, in FBPase reduction, but revealed that NTRC is unable to reduce the enzyme, despite the fact that the light-dependent reduction of FBPase is severely affected in the *ntrc* mutant (Ojeda et al., 2017). Therefore, these studies show that the activity of NTRC is needed for the function of different chloroplast Trxs; though for the redox regulation of FBPase the effect of NTRC seems to be exerted indirectly.

## The Central Role of 2-Cys Prxs: Integrating Antioxidant and Redox Regulatory Mechanisms

**Figure 3:**
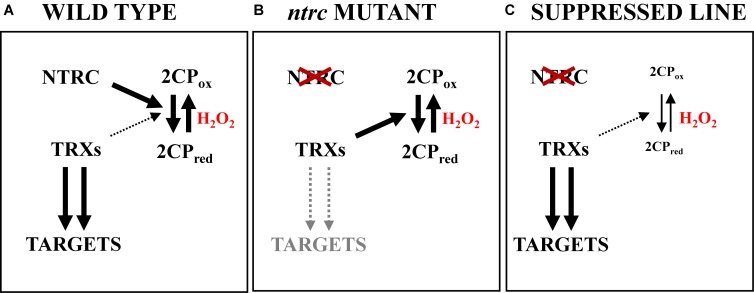
Low contents of 2-Cys Prxs exert a suppressor effect of the *ntrc* phenotype. **(A)** In chloroplasts from wild type plants, NTRC, which is the most efficient reductant of 2-Cys Prxs, maintains the redox state of these enzymes. Consequently, drainage of electron equivalents from the pool of Trxs is negligible and, thus, the redox state of Trxs is appropriate for the light-dependent reduction of redox-regulated targets. **(B)** In the *ntrc* mutant, the lack of the most efficient reductant of 2-Cys Prxs causes the imbalance of the redox state of these enzymes, which accumulate in oxidized form. Though Trxs transfer electrons to 2-Cys Prxs less efficiently than NTRC, the accumulation of the oxidized form of 2-Cys Prxs provokes a significant drainage of reducing equivalents from the pool of Trxs, thereby affecting the light-dependent reduction of downstream targets. **(C)** The suppressor effect is produced by decreased levels of 2-Cys Prxs. In the absence of NTRC, 2-Cys Prxs accumulate in oxidized form, however, as the amount of 2-Cys Prxs is low, the drainage of reducing equivalents from the pool of Trxs is also low, and hence the redox state of the pool of Trxs is appropriate for the reduction of downstream targets.

The puzzling question arising is how NTRC exerts such pleiotropic effects on different chloroplast redox regulated processes while, at least for the light-dependent redox regulation of FBPase, the effect is exerted without the direct interaction of both enzymes. The clarification of this conundrum came from the analysis of Arabidopsis lines combining NTRC and 2-Cys Prxs mutations. Although these plants are devoid of the redox regulatory function of NTRC, the phenotype of these mutants resembles that of the wild type (Pérez-Ruiz et al., 2017). This counterintuitive result indicated that the deficiency of 2-Cys Prxs exerts a suppressor effect of the *ntrc* mutant phenotype. Moreover, the overexpression of 2-Cys Prxs in the *ntrc* mutant background provoked the aggravation of the growth inhibition phenotype of these transgenic plants while the overexpression of the 2-Cys Prxs in the wild type background exerted no or low effect (Pérez-Ruiz et al., 2017). These results indicated that the increase of the content of 2-Cys Prxs becomes toxic for plant growth only when NTRC is not present. Although the absence of NTRC is expected to severely impair the antioxidant capacity of 2-Cys Prxs, it is important to take into account that, as stated above, different chloroplast Trxs are able to transfer reducing equivalents to 2-Cys Prxs, albeit much less efficiently than NTRC (Broin et al., 2002; Collin et al., 2003, 2004; Dangoor et al., 2012; Eliyahu et al., 2015; Hochmal et al., 2016; Vaseghi et al., 2018; Yoshida et al., 2018). Indeed, the light-dependent reduction of 2-Cys Prxs observed in the *ntrc* mutant (Pérez-Ruiz et al., 2017) suggests that these enzymes deplete reducing power from the pool of Trxs. Based on these results, it was hypothesized that in wild type plants the redox state of 2-Cys Prxs is maintained by NTRC, which is the most efficient reductant of the enzyme. Thus, the drainage of reducing equivalents from the other chloroplast Trxs would be very low and, consequently, the redox state of the pool of Trxs allows the light-dependent redox regulation of downstream targets (Figure 3A). In contrast, the *ntrc* mutant lacks the major source of reducing equivalents for 2-Cys Prxs, hence causing the accumulation of the oxidized form of these enzymes. Despite the lower efficiency of electron transfer of Trxs, the increased level of oxidized 2-Cys Prxs in plants lacking NTRC would provoke higher drainage of reducing equivalents from the pool of Trxs, compromising the light-dependent reduction of downstream targets (Figure 3B). Finally, in plants combining NTRC and 2-Cys Prx mutations, the decreased levels of 2-Cys Prxs would alleviate the drainage of reducing equivalents from the pool of Trxs, hence keeping sufficiently reduced the stromal Trxs for the light-dependent reduction of downstream targets, which would be thus the molecular basis of the suppression of the *ntrc* phenotype (Figure 3C). The analysis of the redox state of Trxs *f*, FBPase and PRK in the suppressed line fully confirmed this hypothesis hence uncovering the central function of 2-Cys Prxs in chloroplast redox regulation (Pérez-Ruiz et al., 2017). Moreover, the suppressed line also recovered wild type levels of chlorophyll biosynthesis enzymes (Richter et al., 2018), indicating that the suppressor effect caused by decreased levels of 2-Cys Prxs is exerted beyond the enzymes of the CBC. Based on these results, we proposed a model according to which the redox balance of 2-Cys Prxs, which is maintained by NTRC and NADPH, modulates the light-dependent reduction of redox regulated enzymes based on Fdx reduced by the photosynthetic electron transport chain, which is fueled to stromal Trxs via the action of FTR (Pérez-Ruiz et al., 2017). This model integrates the redox exchange of Trx and redox-regulated targets with hydrogen peroxide via the action of 2-Cys Prxs.

As mentioned above, the simultaneous lack of NTRC and Trx *x* or Trxs *f* cause dramatic loss of photosynthetic efficiency, light-dependent redox regulation of CBC enzymes and, consequently, severe growth inhibition phenotypes (Ojeda et al., 2017). Thus, to test the robustness of the function of the couple NTRC-2-Cys Prxs in chloroplast redox regulation, mutants plants deficient in NTRC, and Trxs *x* or *f* were combined with decreased contents of 2-Cys Prxs (Pérez-Ruiz et al., 2017; Ojeda et al., 2018b). Decreased contents of 2-Cys Prxs recovered growth phenotypes in plants lacking NTRC and Trx *x* (Ojeda et al., 2018b) or NTRC and Trxs *f* (Pérez-Ruiz et al., 2017), thus extending the suppressor effect of the deficiency of 2-Cys Prxs to the dramatic growth inhibition phenotypes caused by the simultaneous lack of NTRC and Trxs. Altogether, these results confirm the essential function of the NTRC-2-Cys Prxs redox couple in chloroplast performance and plant growth.

## 2-Cys Prxs Participate in Chloroplast Enzyme Oxidation in the Dark

The redox regulation of chloroplast enzymes in response to light was discovered in the sixties of the past century; since then, most studies have focused on the light-dependent enzyme activation by reduction, achieving a comprehensive knowledge of this regulatory mechanism (Buchanan, 2016). At the same time, it became clear that redox regulated enzymes of the CBC such as FBPase (Leegood and Walker, 1980) and glyceraldehyde phosphate dehydrogenase (GAPDH) (Sparla et al., 2002) were rapidly oxidized in the dark; however, the mechanism of enzyme oxidation has remained unknown. Our model of chloroplast redox regulation proposes a central role of 2-Cys Prxs in maintaining the redox state of the pool of Trxs that participate in the light-dependent redox regulation of chloroplast metabolic pathways including enzymes of the CBC (Pérez-Ruiz et al., 2017; Ojeda et al., 2018b) and the chlorophyll biosynthesis pathway (Richter et al., 2018). An important consequence of this model is that hydrogen peroxide acts as sink of reducing equivalents and, thus, could be an effective way of relieving reducing equivalents of the pool of Trxs in the dark, hence providing a possible explanation for the long-standing question of how chloroplast enzymes become oxidized in the night. Our group addressed this possibility by analyzing the redox state of well-studied redox-regulated enzymes, FBPase, GAPDH and the γ subunit of ATPase in the Arabidopsis *2cpab* mutant, which is knock out for 2-Cys Prxs A and B (Ojeda et al., 2018a). The enzymes under analysis were detected almost fully reduced in both wild type and *2cpab* mutant plants adapted to light. While in the wild type these enzymes were rapidly oxidized in the dark, oxidation was significantly delayed in the *2cpab* mutant, indicating the participation of 2-Cys Prxs in the short-term oxidation of chloroplast enzymes in the dark (Ojeda et al., 2018a). Independently, Vaseghi et al. (2018) reported the participation of 2-Cys Prxs in the oxidation of reductively activated chloroplast enzymes FBPase, PRK, and NADPH-dependent malate dehydrogenase (MDH) and showed that oxidation was compromised in Arabidopsis mutant plants devoid of 2-Cys Prxs. The analysis of the oxidative activity of the Trx-like2 (Trx-L2) protein *in vitro*, in conjunction with the use of a severe 2-Cys Prxs knock down mutant of Arabidopsis led Hisabori’s group to propose the participation of an oxidant pathway formed by Trx-L2/2-Cys Prxs in enzyme oxidation in the dark (Yoshida et al., 2018). The identification of the function of 2-Cys Prxs in the oxidation of thiol groups is in line with the previous proposal of the oxidizing activity of this enzyme in response to moderate light intensity (Dangoor et al., 2012) and for oxidation of the small subunit of AGPase (Eliyahu et al., 2015).

**Figure 4:**
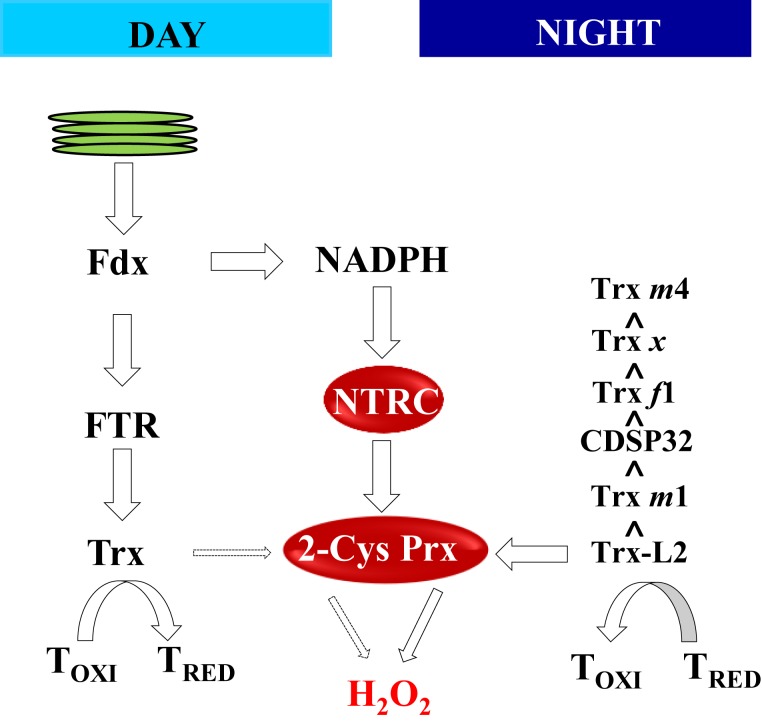
The NTRC-2-Cys Prx couple controls the reduction/oxidation balance of redox-regulated targets in the day and in the night. During the day, photo-reduced Fdx fuels reducing equivalents via FTR and Trxs for the reduction (activation) of redox-regulated enzymes. The redox state of the pool of Trxs is maintained by the couple NTRC-2-Cys Prxs, which relies on NADPH as source of reducing power. In the dark, the input of reducing equivalents via reduced Fdx ceases and Trxs mediate the oxidation of reduced targets transferring electrons through the activity of 2-Cys Prxs to hydrogen peroxide, which acts as final sink of electrons. The oxidant efficiency of different Trxs is presented as proposed by Yoshida et al. (2018) and Vaseghi et al. (2018).

Therefore, after decades of research focused on the mechanisms leading to enzyme activation in the light, three independent studies (Ojeda et al., 2018a; Vaseghi et al., 2018; Yoshida et al., 2018) identified the participation of 2-Cys Prxs in enzyme oxidation in the dark. The characterization of the 2-Cys Prx interactome revealed the interaction of 2-Cys Prxs with a large number of chloroplast proteins including FBPase (Cerveau et al., 2016). Therefore, a possibility to be taken into account is that FBPase oxidation occurs by the direct transfer of electrons from the enzyme to 2-Cys Prxs. However, studies performed *in vitro* with recombinant enzymes (Ojeda et al., 2018a; Yoshida et al., 2018) or with extracts from chloroplast stroma (Vaseghi et al., 2018) showed that reduced FBPase was not directly oxidized in the presence of 2-Cys Prx or Trxs. In contrast, the addition of 2-Cys Prxs and Trxs resulted in FBPase oxidation (Ojeda et al., 2018a), indicating the participation of these Trxs in the process of oxidation. This notion was further supported by Vaseghi et al. (2018), who determined the MDH oxidation efficiency by different Trxs, including canonical *m*- *f*- and *x*-types and Trx-like proteins such as chloroplast drought-induced stress protein of 32 kDa (CDSP32). Finally, it was shown that Trx-L2, which has the less negative reducing potential and is unable to reduce FBPase, is the most efficient chloroplast Trx for transfer of reducing equivalents of reduced enzymes to 2-Cys Prxs and H_2_O_2_ (Yoshida et al., 2018). Altogether, these studies proposed a pathway of oxidation involving Trxs, which would act as intermediates between the reduced targets and 2-Cys Prxs (Figure 4). This finding is an important advance in the current understanding of the control of chloroplast photosynthetic metabolism by light and darkness. However, it should be noted that, albeit delayed, enzyme oxidation still takes place in mutant plants lacking 2-Cys Prxs, indicating the participation of additional mechanism(s) in the process of enzyme oxidation in the dark. In search of these additional mechanisms, our group tested the participation of Prx Q and Prx IIE, monomeric Prxs present in Arabidopsis chloroplasts (Dietz, 2011). However, Arabidopsis triple mutants combining the lack of 2-Cys Prxs with severely decreased levels of either Prx Q or Prx IIE showed similar growth phenotype and rates of enzyme oxidation in the dark than the *2cpab* mutant, suggesting that these Prxs have no relevant function in enzyme oxidation (Ojeda et al., 2018a).

## Concluding Remarks and Future Prospect

While redox regulation in heterotrophic organisms involves a low number of Trxs and NTRs, and depends on NADPH, redox regulation in chloroplasts involves two redox systems: NTRC, which relies on NADPH, and the FTR-Trxs system, in which a large set of Trxs relies on photo-reduced Fdx. Therefore, redox regulation in chloroplasts is much more complex than in heterotrophic organisms, probably reflecting the sessile life style of plants and their necessity to rapidly respond to natural fluctuations in light. A recent report has identified the central role of 2-Cys Prxs integrating disulfide-dithiol exchange of redox-regulated enzymes and hydrogen peroxide detoxification (Pérez-Ruiz et al., 2017). Moreover, the participation of 2-Cys Prxs in the short-term oxidation of chloroplast enzymes in the dark has been recently reported (Ojeda et al., 2018a; Vaseghi et al., 2018; Yoshida et al., 2018), thus starting to solve the long-lasting question of the mechanism that allows the rapid oxidation of chloroplast enzymes in the night (Jacquot, 2018). Nevertheless, the participation of additional mechanism(s), yet to be identified, as well as the role of chloroplast Trxs in the oxidation process need to be clarified. Finally, the function of NTRC could be exclusively the maintenance of the redox balance of 2-Cys Prxs for proper chloroplast function, however whether this enzyme hase additional regulatory functions deserves further attention in next few years.

## Author Contributions

FJC designed and wrote the initial version of the manuscript. VO, VD-R, MG, and JMP-R contributed to the final version.

## Conflict of Interest Statement

The authors declare that the research was conducted in the absence of any commercial or financial relationships that could be construed as a potential conflict of interest.
